# Risk of Lung Cancer in Workers Exposed to Benzidine and/or Beta-Naphthylamine: A Systematic Review and Meta-Analysis

**DOI:** 10.2188/jea.JE20150233

**Published:** 2016-09-05

**Authors:** Kimiko Tomioka, Keigo Saeki, Kenji Obayashi, Norio Kurumatani

**Affiliations:** 1 Nara Prefectural Health Research Center, Nara Medical University, Kashihara, Japan; 1 奈良県立医科大学県民健康増進支援センター; 2 Department of Community Health and Epidemiology, Nara Medical University, Kashihara, Japan; 2 奈良県立医科大学地域健康医学教室

**Keywords:** benzidine, beta-naphthylamine, lung cancer, occupational exposure, systematic review, ベンジジン, β‐ナフチルアミン, 肺癌, 職業性曝露, システマティック・レビュー

## Abstract

Benzidine (BZ) and beta-naphthylamine (BNA) have been classified as definite human carcinogens for bladder cancer by the International Agency for Research on Cancer. However, the epidemiological evidence for an association between exposure to BZ and/or BNA and lung cancer has been inconclusive. We conducted a systematic review and meta-analysis to determine the risk for lung cancer among workers exposed to BZ/BNA. A systematic literature search was conducted to identify studies that had reported occupational BZ/BNA exposure and the outcome of interest (lung cancer death and/or incidence). Meta-analyses were performed using random effects models to combine standardized mortality ratios (SMRs) or standardized incidence ratios (SIRs). We identified 23 retrospective cohort studies including 1745 cases of lung cancer; only one study reported smoking-adjusted lung cancer risk. A significantly increased lung cancer risk (pooled SMR/SIR 1.28; 95% CI, 1.14–1.43) was observed by combining all studies, with significant heterogeneity among studies (I^2^ = 64.1%, *P* < 0.001). Effect estimates were higher for studies with direct BZ/BNA exposure (ie, dyestuff and manufacturing industries) (pooled SMR/SIR 1.58; 95% CI, 1.31–1.89), and studies that identified BZ/BNA-associated bladder cancer with SMR/SIR ≥4.7 (pooled SMR/SIR 1.68; 95% CI, 1.35–2.09). Effect estimates were similar for studies with and without concomitant occupational exposure to chromium, asbestos, arsenic, or bis(chloromethyl) ether. The cumulative meta-analysis showed that the evidence of association between occupational BZ/BNA exposure and lung cancer has been stable since 1995. Although the results of this meta-analysis have the potential for confounding by smoking and heterogeneity, our findings suggest that a finding of lung cancer following occupational BZ/BNA exposure should be considered to be a potential occupational disease.

## Abstract (ja)

ベンジジン、β‐ナフチルアミンは国際がん研究機関によって人に膀胱癌を引き起こす明らかな発がん物質であると分類されている。しかし、ベンジジン、β‐ナフチルアミンの曝露と肺癌との関連については十分検討されていない。我々はベンジジン、β‐ナフチルアミンに曝露された労働者における肺がんリスクを評価することを目的にシステマティック・レビューおよびメタ･アナリシスを行った。職業性ベンジジン、β‐ナフチルアミン曝露と関心のあるアウトカム（肺がんによる死亡や罹患）を報告した研究を同定するために系統的文献検索を実施した。メタ･アナリシスはランダム効果モデルを用いて標準化死亡比や標準化罹患比を結合させた。1,745の肺がん症例を含む23の歴史的コホート研究を同定した；喫煙を調整した肺がんリスクを報告していた研究は1つだけであった。すべての研究を結合することによって、有意な異質性を伴った（I^2^ = 64.1%, p <0.001）有意な肺がんリスクの上昇が認められた（pooled SMR/SIR 1.28; 95% CI, 1.14-1.43）。効果の推定値はベンジジン、β‐ナフチルアミンの直接曝露を伴った研究（すなわち、染料や製造産業）（pooled SMR/SIR 1.58; 95% CI, 1.31-1.89）やベンジジン、β‐ナフチルアミンに関連した膀胱癌のSMR/SIRが4.7以上の研究（pooled SMR/SIR 1.68; 95% CI, 1.35-2.09）で高くなっていた。効果の推定値はクロム、アスベスト、ヒ素、ビスクロロメチルエーテルの職業性曝露を伴っている研究と伴っていない研究では同じであった。累積メタ・アナリシスを行った結果、職業性ベンジジン、β‐ナフチルアミン曝露と肺癌との関連は1995年時点で確立していた。このメタ･アナリシスの結果は喫煙や異質性によって交絡されている可能性があるが、我々の発見は職業性ベンジジン、β‐ナフチルアミン曝露を伴う肺癌は職業癌の可能性を考慮すべきであることを示唆している。

## INTRODUCTION

Benzidine (BZ) and beta-naphthylamine (BNA), which are aromatic amines, are classified by the International Agency for Research on Cancer (IARC) as definite human carcinogens (Group 1) on the basis of sufficient evidence of bladder cancer in animals and human beings.^1^^,^^2^ Because of their carcinogenicity, the manufacture and use of BZ and BNA have been prohibited in most advanced countries.^3^^,^^4^ However, the production and use of BZ and BNA have been reported in some developing countries,^5^ occupational exposure to BZ and/or BNA still occurs in laboratory research and diagnostic testing,^1^^,^^2^ and some aromatic amines that have been used as substitutes can metabolize in the body to BZ or BNA.^2^^,^^6^^,^^7^ Therefore, occupational exposure to BZ and/or BNA is an ongoing issue.

In addition to the carcinogenicity of BZ and BNA on the bladder, some occupational epidemiological studies have observed carcinogenic risks at sites besides the bladder (eg, lungs,^8^^–^^11^ esophagus,^8^ liver, gallbladder, bile duct,^12^ intestines, larynx,^13^ and lymphohematopoie^14^).^11^ However, the epidemiological evidence for BZ- and/or BNA-induced cancers other than in the bladder is not as strong. This is due to inconsistencies in tumor site studies.^11^

Some of the studies that examined cancer risk from occupational exposure to BZ and/or BNA have identified a significantly increased risk of lung cancer.^8^^–^^11^ However, most studies^8^^–^^10^ failed to confirm a positive dose-response relationship between BZ/BNA exposure and the risk for lung cancer due to an insufficient number of cases. The inadequate statistical evidence of those individual studies would not allow for a proper interpretation of the effect of BZ and/or BNA in regards to lung cancer. Additionally, to our knowledge, there have been no meta-analyses of lung cancer risk in workers exposed to BZ and/or BNA.

To examine whether occupational exposure to BZ and/or BNA is associated with the risk of lung cancer, we conducted a systematic review and meta-analysis using data from occupational epidemiological studies regarding the association of BZ and/or BNA with lung cancer risk.

## METHODS

This systematic review and meta-analysis was conducted following the Meta-analysis of Observational Studies in Epidemiology (MOOSE) guidelines^15^ and was reported in accordance with the Preferred Reporting Items for Systematic Reviews and Meta-Analyses (PRISMA) statement.^16^ This systematic review protocol was registered with the International Prospective Register of Systematic Reviews (PROSPERO) database (registration number: CRD42014010250) and published in an academic journal.^17^ This systematic review and meta-analysis is based on a method of prospective systematic reviews.^18^

### Participants

We included subjects with unequivocal evidence of occupational exposure to BZ and/or BNA, such as dyestuff workers, workers from BZ/BNA manufacturing plants, leather tannery workers who used BZ-based dyes, and rubber industry workers exposed to BZ/BNA present as a contaminant in antioxidants used in manufacturing. Subjects who worked at the same factories but were not exposed to either BZ or BNA were excluded.

### Study designs

Eligible studies were comparative observational studies that reported occupational BZ/BNA exposure and the outcome of interest (lung cancer death and/or lung cancer incidence). Retrospective cohort studies (also known as historical cohort studies), prospective cohort studies, and case-control studies were included in this review.

### Exposures

For cohort studies, ascertainment of exposure to BZ and/or BNA was based on written records of exposure measurements or work history. For case-control studies, BZ and/or BNA exposure was ascertained by secure records (eg, surgical records), structured interviews with blinding to case/control status, interviews without blinding to case/control status, or written self-reports.

### Comparators

For cohort studies, use of a comparator was not a requirement for inclusion. For case-control studies, the control group must have included subjects who had no history of lung cancer.

### Outcomes

Our outcomes were lung cancer death and/or lung cancer incidence based on clinically confirmed diagnoses (ie, death certificates, cancer registry or other national recording system, or hospital or doctor’s records). Effect measures included standardized mortality ratio (SMR), standardized incidence ratio (SIR), and odds ratio (OR) for the association between BZ/BNA exposure and lung cancer risk. SMRs and SIRs were based on an external comparison group (ie, national or regional population), and ORs were based on a population- or hospital-based control group.

### Search strategy

The search strategies were carried out by the research team and an expert librarian (TS). No language restriction was enforced, conditional to the provision of an English abstract. A date restriction was not imposed. A comprehensive search of databases from each database’s earliest inclusive dates to September 11, 2014, was conducted; a subsequent update search (to March 19, 2015) was also conducted. The databases included MEDLINE, Excerpta Medica DataBase (EMBASE), and Cumulative Index to Nursing and Allied Health Literature (CINAHL). Search terms included controlled vocabulary and text-words, and details of the search strategy for MEDLINE are provided in eTable 1. Additional studies were identified from the reference list of articles and relevant reviews. Furthermore, we contacted the authors of the studies we included, asking them for data about other published or unpublished works our search had not found.

### Study selection

We assessed for inclusion all titles and abstracts identified during the literature search. Two of the authors (KT and KS) independently examined the search results for potentially eligible studies. Disagreements were resolved through consultation with a third author (KO). For studies that appeared to meet the criteria for this review, we obtained and examined the full-text articles. If multiple reports of the same study were encountered, they were used only once; the record containing the most data (for example, greatest sample size, or longest follow-up period) was used. This usually meant using the most recently published reference, but if lung cancer risk data was only noted an earlier paper, that information was used.^19^ For studies reporting data on incidence and mortality, incidence data were selected.

### Data extraction

Two of the authors (KT and KS) independently extracted study details from the full text articles using a pilot-tested form. When discrepancies arose, a third author (KO) negotiated a consensus. We abstracted data on the number of observed deaths or cases, the number of expected deaths or cases, and the effect measure and the 95% confidence interval (CI) for lung cancer (essential) and bladder cancer (if available). We collected data on bladder cancer to assess heterogeneity between the included studies. For the studies in which CIs were not reported, we calculated them by the exact probabilities of the Poisson distribution using the observed deaths/cases and expected deaths/cases reported in the articles.^20^ If a study used both national and regional populations to compute the expected deaths/cases, the regional results were used, because a regional population of study subjects is preferable to the national population for controlling geographic differences in disease incidence.^21^ Data on the following study characteristics were also extracted where available: year of publication, proportion of males, duration of follow-up, duration of employment, country and geographic area, industry type, occupational exposure to chemicals (including BZ and BNA), information on cigarette smoking, and information relating to quality assessment. Also, information concerning national lung cancer incidence rates were obtained from GLOBOCAN 2012 estimates,^22^ and data on national prevalence of cigarette smoking was obtained from the World Health Organization Report on the Global Tobacco Epidemic 2013.^23^

### Quality assessment

Quality was assessed by two reviewers (KT and KS), and discrepancies were resolved by consulting with a third reviewer (KO). For case-control studies, we used the Newcastle-Ottawa Scale (NOS).^24^ This assessment scale consists of eight items that are categorized into three major components: selection, comparability, and exposure. For cohort studies, a modified version of the NOS^19^ was used. This modified NOS was developed for assessing the quality of occupational cohort studies and includes five quality components: representativeness of the exposed cohorts, exposure assessment/reporting, comparability of exposed and non-exposed cohort, assessment of outcomes, and adequacy of follow-up.

### Data synthesis

We performed meta-analysis to obtain the weighted average (pooled) of the SMR and SIR for cohort studies and the OR for case-control studies using the Comprehensive Meta-Analysis version 2.0 software (Biostat, Englewood, NJ, USA). Effect measures were pooled using random-effects models, which were weighted using the inverse of the variance.^25^ The presence of between-study heterogeneity was assessed using the I^2^ test.^26^ Publication bias was also examined visually using funnel plots and mathematically using the Egger regression asymmetry test.^27^

In order to explain any heterogeneity seen between studies, we used subgroup analyses. The covariates considered were study outcome (incidence vs mortality), study area (Asia vs Europe vs the United States), reference group (national vs regional), cohort/sample size, type of industry, type of exposure to BZ/BNA, occupational exposure to some carcinogen for lung cancer (no vs yes), year of starting the production/use of BZ/BNA, follow-up duration, SMR/SIR for bladder cancer, national incidence rate for lung cancer, and national prevalence of cigarette smoking. The covariates with continuous variables were divided into two subgroups using the median. High SMR/SIR for bladder cancer was used as the proxy measure for probable higher exposure to BZ/BNA. Based on the information on type of industry, the situation of exposure to BZ/BNA was dichotomized into direct (dye and manufacture) and indirect (leather tanning and rubber).

In our sensitivity analyses, we separately analyzed category outcomes of the assessed study quality variables to determine if there was any relationship between quality and outcome. Additionally, we assessed the influence individual studies had on the pooled estimate by deleting all risk estimates of each study from the meta-analysis and determining the range of pooled estimates. We also conducted a cumulative meta-analysis in publication year order to determine just when the risk estimate became statistically significant and to clarify any variations.^28^

Instead of a dose-response analysis, we did a meta-analysis that combined the results of workers who had the highest occupational BZ/BNA exposure from studies reporting data relevant to level of exposure to BZ/BNA.^29^

## RESULTS

### Description of included studies

**Figure 1.**
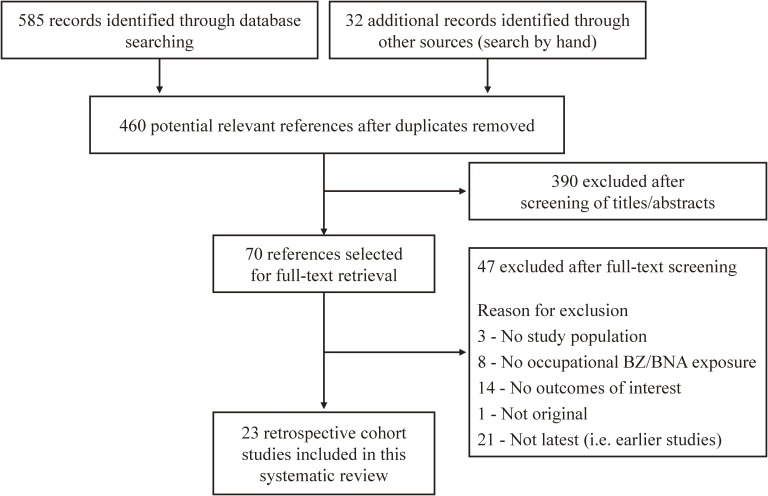
Flow diagram for selection of studies included in the systematic review.

Our search identified 460 candidate references (Figure 1). After the title and abstract assessment, 390 of those articles were excluded. After full-text assessment, we determined that 23 studies met the inclusion criteria, and we included these studies in this analysis.^8^^–^^14^^,^^30^^–^^45^ We could identify neither prospective cohort studies nor case-control studies that examined the association between lung cancer and occupational exposure to BZ/BNA. Therefore, all the included studies were retrospective cohort studies. Table 1 summarizes the main characteristics of the cohort studies investigated in this meta-analysis (the papers are described in detail in eTable 2, eTable 3, and eTable 4).

**Table 1.** Characteristics of 23 cohort studies included in a meta-analysis of lung cancer and exposure to benzidine (BZ) and/or beta-naphthylamine (BNA)

ID	Reference (first author and year of publication)	Type of industry	Type of AA	Country	Cohort size^a^	% male^b^	Smoking information
1	Fox, 1976^30^	Rubber	BNA	UK	12 781	100	No
2	Delzell, 1982^31^	Rubber	BNA	USA	2666	100	No
3	Morinaga, 1982^12^	Manufacturing	Mixed	Japan	244	NA	No
4	Gustavsson, 1986^32^	Rubber	BNA	Sweden	3000^c^	65	No
5	Costantini, 1989^33^	Tannery	BZ	Italy	2926	100	No
6	Delzell, 1989^34^	Dye	BZ	USA	379	100	No
7	Sorahan, 1989^35^	Rubber	BNA	UK	15 206	100	No
8	Chen, 1990^36^	Tannery	BZ	China	901	100	Yes
9	Morinaga, 1990^37^	Manufacturing	Mixed	Japan	794	100	No
10	You, 1990^38^	Dye	BZ	China	550	76.2	Yes
11	Bulbulyan, 1995^8^	Dye	Mixed	Russia	514	52.6	No
12	Naito, 1995^39^	Dye	Mixed	Japan	356	98.9	No
13	Sitarek, 1995^40^	Dye	BZ	Poland	1500^c^	81.8	No
14	Szeszenia-Dąbrowska, 1995^41^	Rubber	Mixed^d^	Poland	6978	100	No
15	Montanaro, 1997^42^	Tannery	BZ	Italy	1244	69.9	No
16	Axtell, 1998^9^	Dye	Mixed^d^	USA	1314	94.9	No
17	Cassidy, 2003^10^	Manufacturing	BNA	USA	374	93.5	Yes^e^
18	Stern, 2003^43^	Tannery	BZ	USA	2000^c^	75.6	No
19	Rosenman, 2004^14^	Dye	BZ	USA	285	90.7	No
20	Mikoczy, 2005^44^	Tannery	BZ	Sweden	2027	76.2	No
21	Pira, 2010^13^	Dye	Mixed	Italy	590	100	No
22	Brown, 2011^45^	Manufacturing	BZ	USA	847	85	No
23	Tomioka, 2015^11^	Dye	Mixed	Japan	224	92.2	Yes

AA, aromatic amines; NA, not available.

^a^The number of sub-cohorts included in this meta-analysis.

^b^Percentage of males in the total cohort.

^c^Estimated due to a lack of data on the number of sub-cohorts.

^d^Mainly BNA.

^e^Incomplete data.

The meta-analysis included studies that covered 1745 cases of lung cancer. Cohort studies had been carried out in Europe, the United States, and Asia from 1976–2015. The industries investigated included dye production (*n* = 9), leather tanning (*n* = 5), the rubber industry (*n* = 5), and BZ/BNA manufacturing (*n* = 4). Regarding smoking, four of the included studies reported information on cigarette smoking, but only one study^11^ calculated the smoking-adjusted risk for lung cancer. Regarding gender, most of the study subjects were male. Nine studies contained only males, and most cohorts with males and females either excluded females from the analysis or presented risk estimates for males and females combined. Regarding the overall meta-analysis, we used the data for male workers when available. If male data had not been published for the individual studies, the effect estimates for both sexes were used in the overall meta-analysis. For occupational exposure to chemicals other than BZ and BNA (see eTable 2), some cohorts were potentially exposed to chromium,^46^ asbestos,^47^ arsenic,^48^ and bis(chloromethyl) ether,^49^ which are classified by the IARC as “carcinogenic to humans” (Group 1) based on evidence of increased lung cancer in people.

The results of the study quality assessment are shown in eTable 5. Quality assessment indicated that 1) in representing the exposed cohort, 20 studies were rated as being high quality (ie, representative); 2) in regards to exposure, 19 studies had high-quality data (ie, formal exposure records based on work history derived from company records), while no studies reported exposure in terms of work-place measurements; 3) for comparability, 12 studies were rated high quality (ie, the use of standard adjustment methods), while nearly half of the included studies (*n* = 11) did not use appropriate methods according to the criteria defined in the modified NOS; 4) as for outcome assessments, 19 studies were assessed using formal records (ie, cancer registry or death certificates); and 5) regarding follow-up adequacy, over half of the studies (*n* = 12) had nearly complete follow-up (ie, 5% or less of the cohort remain untraced), while 6 studies did not report on loss to follow-up.

### Results of the overall meta-analysis

**Figure 2.**
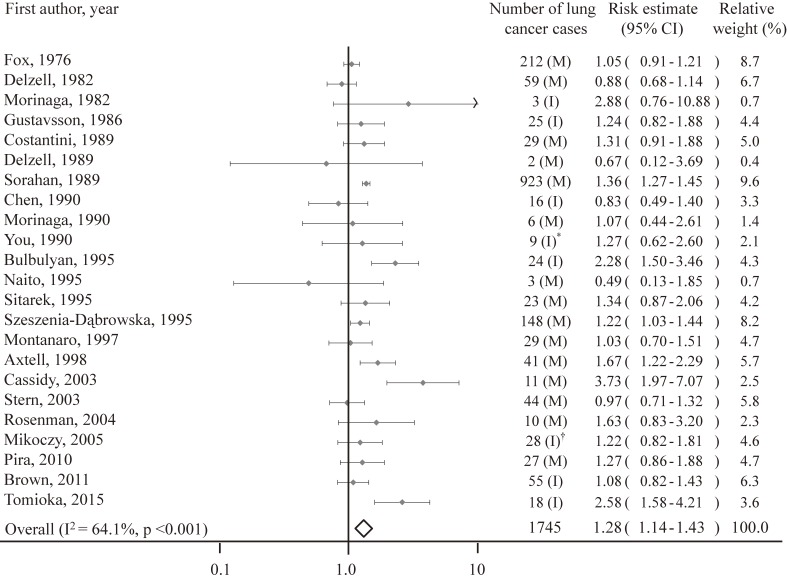
Forest plot of studies included in the meta-analysis of exposure to benzidine and/or beta-naphthylamine and lung cancer: all studies combined. I, incidence; M, mortality. *Respiratory cancer. ^†^Obtained by contacting author.

A forest plot summarizing the results and weights applied to each study is shown in Figure 2. The 23 effect estimates from included studies ranged from 0.49 to 3.73 and resulted in a significantly increased overall pooled risk estimate of 1.28 (95% CI, 1.14–1.43), with significant heterogeneity among studies (I^2^ = 64.1%, *P* < 0.001).

### Subgroup analysis

There was enough heterogeneity evidence that it was decided to investigate possible explanatory factors. Table 2 presents the findings from the subgroup analyses for all covariates. The 8 studies reporting incidence of lung cancer resulted in a pooled risk estimate of 1.41 (95% CI, 1.13–1.75) compared with a pooled estimate of 1.23 (95% CI, 1.07–1.42) from the 15 lung cancer mortality studies. The amount of variation due to heterogeneity (I^2^) was the same for both subgroups (I^2^ = 65.4%). Cohort size, type of industry, situation of exposure to BZ/BNA, and magnitude of the SMR/SIR for bladder cancer were each statistically significant predictors of the pooled risk estimates for lung cancer. Pooled risk estimates were elevated for dyestuff workers (1.60; 95% CI, 1.29–1.99) and workers at BZ/BNA manufacturing plants (1.51; 95% CI, 1.07–2.14), but not for leather tanning workers (1.07; 95% CI, 0.85–1.36) or workers in the rubber industry (1.15; 95% CI, 0.95–1.38). Pooled risk estimates were increased for small cohorts (1.60; 95% CI, 1.29–1.98) compared with large cohorts (1.17; 95% CI, 1.02–1.34); workers with direct exposure to BZ/BNA (1.58; 95% CI, 1.31–1.89) compared with workers with intermittent contact with BZ/BNA (1.12; 95% CI, 0.97–1.29); and cohorts that reported an SMR/SIR for bladder cancer ≥4.7 (1.68; 95% CI, 1.35–2.09) compared with other cohorts (1.15; 95% CI, 1.02–1.31). Pooled risk estimates were similar in workers with occupational exposure to some carcinogens for lung cancer (1.37; 95% CI, 1.11–1.68) and workers without occupational exposure to carcinogens for lung cancer (1.24; 95% CI, 1.07–1.44). No difference in risk was found between cohorts that started the production/use of BZ/BNA before 1945 and cohorts that initiated it after 1945, as well as between studies with high national incidence rates for lung cancer and studies with low rates. In addition, no difference in risk was shown between cohorts with high prevalence of male smoking and cohorts with low prevalence of male smoking. In subgroup analyses, all but four subgroups (dyestuff workers, leather tanning workers, exposure to BZ, and long follow-up duration) showed significant heterogeneity, with I^2^ > 50%.

**Table 2.** Pooled risk estimates resulting from subgroup analyses and sensitivity analyses

	Number of cases	Number of studies	Pooled risk estimate (95% CI)	*P*-value^a^		Heterogeneity	

						I^2^ (%)	*P*-value
Study outcome							
Incidence	178	8	1.41 (1.13–1.75)	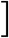	0.312	65.4	0.005
Mortality	1567	15	1.23 (1.07–1.42)			65.4	<0.001
Study area							
Asia	55	6	1.36 (0.96–1.91)	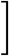	0.931	63.6	0.017
Europe	1468	10	1.28 (1.09–1.50)			55.0	0.018
United States	222	7	1.25 (1.01–1.56)			76.3	<0.001
Reference group							
National	1621	16	1.23 (1.08–1.41)	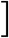	0.214	60.0	0.001
Regional	124	7	1.48 (1.14–1.92)			73.9	0.001
Cohort size							
Large (901–15 206)	1577	12	1.17 (1.02–1.34)	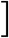	0.015	61.8	0.002
Small (224–847)	168	11	1.60 (1.29–1.98)			63.9	0.002
Type of industry							
Dye	157	9	1.60 (1.29–1.99)	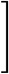	0.035	37.9	0.116
Leather tanning	146	5	1.07 (0.85–1.36)			0.0	0.568
Manufacturing	75	4	1.51 (1.07–2.14)			78.1	0.003
Rubber	1367	5	1.15 (0.95–1.38)			79.3	0.001
Situation of exposure to BZ/BNA (Relisted)							
Direct (dye and manufacture)	232	13	1.58 (1.31–1.89)	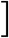	0.004	57.7	0.005
Indirect (tannery and rubber)	1513	10	1.12 (0.97–1.29)			64.6	0.003
Type of exposure to BZ/BNA							
BNA	1230	5	1.24 (0.99–1.55)	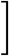	0.100	86.6	<0.001
BZ	245	10	1.13 (0.93–1.37)			0.0	0.784
Mixed	270	8	1.56 (1.25–1.95)			63.3	0.008
(Relisted)							
BNA (BNA and mixed)	1500	13	1.40 (1.19–1.65)			76.6	<0.001
BZ (BZ and mixed)	515	18	1.30 (1.13–1.49)			45.7	0.018
Occupational exposure to some carcinogen for lung cancer^b^							
No	1438	16	1.24 (1.07–1.44)	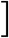	0.455	60.5	0.001
Yes	307	7	1.37 (1.11–1.68)			74.2	0.001
Year of starting the production/use of BZ/BNA							
Early (1900–45)	494	13	1.28 (1.09–1.51)	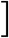	0.981	66.0	<0.001
Late (1946–67)	1251	10	1.28 (1.05–1.56)			64.1	0.003
Follow-up duration							
Long (39–60 years)	1331	11	1.23 (1.05–1.45)	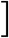	0.452	27.7	0.181
Short (6–38 years)	414	12	1.35 (1.12–1.63)			74.9	<0.001
Bladder cancer SMR/SIR^c^							
High (4.70–38.25)	133	10	1.68 (1.35–2.09)	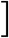	0.003	55.0	0.018
Low (0.58–2.73)	1609	12	1.15 (1.02–1.31)			63.1	0.002
National incidence rate for lung cancer in 2012^d^							
High (44.2–60.5)	442	12	1.29 (1.09–1.53)	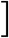	0.924	69.9	<0.001
Low (19.4–38.8)	1303	11	1.27 (1.07–1.52)			57.7	0.009
Prevalence of male smoking in 2011^e^							
High (31–59)	335	12	1.34 (1.12–1.62)	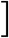	0.506	51.9	0.018
Low (21–25)	1410	11	1.24 (1.05–1.46)			73.9	<0.001
Sensitivity analyses^f^							
Representativeness: representative	1703	20	1.24 (1.10–1.39)			61.2	<0.001
Exposure measurement: formal	1666	19	1.24 (1.09–1.42)			63.1	<0.001
Comparability of groups: standard	1345	12	1.35 (1.17–1.55)			61.2	0.003
Assessment of outcome: formal	1681	19	1.28 (1.13–1.46)			69.6	<0.001
Adequacy of follow-up: virtually complete	1340	12	1.26 (1.05–1.50)			72.0	<0.001

^a^Between subgroups.

^b^Chrome, asbestos, arsenic, and bis(chloromethyl) ether.

^c^Data not available in the Morinaga (1982) study.

^d^Age-standardized rates per 100 000 men.

^e^Age-standardized estimated prevalence of smoking among males aged 15 and older.

^f^Pooled risk estimate obtained after excluding all studies but those rated as having the highest grade for each quality component individually.

### Sensitivity analysis

#### Study quality

Through subgroup analyses, we tried to determine if study quality influenced outcome. The bottom of Table 2 presents the results of omitting all studies but those of the highest quality for each separate quality component. Results remained robust after exclusion of those studies perceived to be of lower quality.

#### Influence of individual studies

Sensitivity analysis by omitting each study in turn had no effect on the meta-analysis results and showed robust results (see eFigure 1). The pooled risk estimate ranged from 1.24 to 1.31, and all pooled risk estimates were statistically significant.

### Assessing publication bias

Visual examination of the funnel plot (see eFigure 2) used to determine publication bias revealed no systematic relation between study size and magnitude of the estimator (SMR/SIR). Likewise, the Egger test did not show significant funnel plot asymmetry (intercept, −0.02; 95% CI, −1.09 to 1.05; one-tailed *P*-value = 0.48).

### Cumulative meta-analysis

**Figure 3.**
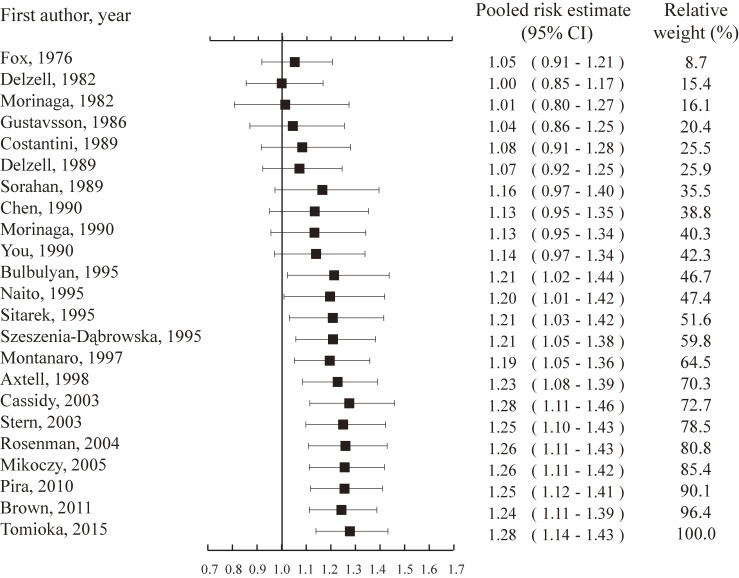
Forest plot of cumulative meta-analysis of lung cancer and occupational exposure to benzidine and/or beta-naphthylamine. Pooled risk estimates (with 95% confidence intervals) by year of publication of subsequent reports. Vertical solid lines = no effect.

The results of the cumulative meta-analysis are depicted in Figure 3. The pooled risk estimate was 1.21 (95% CI, 1.02–1.44) in 1995, when the first 10 cohort studies had been published. Although 12 additional cohort studies have been published since, the summary estimates have stayed basically steady, and these results have remained significant until the end of the analysis.

### Analysis of highly exposed groups

We identified 6 studies that evaluated the risk estimates of exposure levels among BZ/BNA-exposed workers (see eTable 6). The pooled lung cancer risk estimate, based on these six high-exposure groups, was 2.33 (95% CI, 1.31–4.14). We found significant heterogeneity across all studies (I^2^ = 67.2%, *P* = 0.009).

## DISCUSSION

We conducted a systematic review and meta-analysis to investigate the association between occupational BZ/BNA exposure and risk of lung cancer. A total of 23 retrospective cohort studies of 1745 cases were included in the analysis. We found a significantly increased risk of lung cancer among workers exposed to BZ/BNA occupationally, with an overall pooled risk estimate of 1.28 (95% CI, 1.14–1.43). Results from cumulative meta-analysis and sensitivity analysis and an absence of publication bias suggest that the results are reliable and robust.

A major finding here is that the pooled risk estimate for lung cancer was elevated both in those studies in which BZ/BNA-associated bladder cancer relative risks were also elevated (pooled risk estimate 1.68; 95% CI, 1.35–2.09) and in those studies in which workers had many opportunities for direct exposure to BZ/BNA (pooled risk estimate 1.58; 95% CI, 1.31–1.89). The results from this analysis of highly exposed workers also indicate a stronger effect than among all workers combined (pooled risk estimate 2.33; 95% CI, 1.31–4.14). In this meta-analysis, no studies reported exposure as work-place measurements, which are the most accurate and unbiased occupational exposure estimates. However, since BZ/BNA exposures are generally apt to be higher in studies finding elevated bladder cancer rates, the presence of a positive bladder cancer finding may be a legitimate substitute for high BZ/BNA exposure. In meta-analysis of cancer risk in occupational settings, a similar approach has been adopted for the purpose of identifying a highly exposed subgroup.^29^^,^^50^ For example, in a meta-analysis of occupational exposure to asbestos and ovarian cancer, the SMR for lung cancer was employed.^29^ Additionally, workers with direct exposure to BZ/BNA (eg, dyestuff workers and workers at BZ/BNA manufacturing plants) are likely to have been exposed to higher concentrations of BZ/BNA than workers exposed to BZ/BNA indirectly (eg, leather tannery workers and rubber industry workers). Hence, these results provide more evidence that the positive findings in this meta-analysis are due to BZ/BNA exposure. Nevertheless, in addition to higher BZ/BNA exposure, reporting a high risk (risk estimate ≥4.7) of bladder cancer may indicate greater confounding by smoking. Therefore, the possibility of a confounding effect from smoking in the studies identifying increased risks of bladder cancer should be considered.

It is not known just how BZ and BNA cause lung cancer, but there is data for a number of possible mechanisms. As with many other aromatic amines, BZ and BNA must be metabolized into reactive electrophiles that react with DNA so they can become carcinogenic.^51^ While some research has found variances in the metabolic patterns of monoarylamines, such as BNA, and diarylamines, such as BZ,^52^ the main chemical reactions needed in BZ and BNA metabolism are the same; initial activation occurs in the liver, and further metabolism that creates more reactive compounds occurs in the bladder.^51^^,^^53^ This is why bladder cancer is the main effect associated with BZ/BNA exposure. The initial activation of aromatic amines via N-oxidation is mediated primarily by cytochrome P4501A2 (CYP1A2).^53^ A prior study did not detect CYP1A2 in most human extrahepatic tissue.^54^ Further metabolism of aromatic amines involves O-acetylation, which is regulated by the enzymes N-acetyltransferases 1 (NAT1).^55^ NAT1 are detected not only in the human urinary bladder^56^ but also in the human peripheral lung.^57^ Although aromatic amine N-oxidation does not take place in the lung, these compounds could undergo N-oxidation in the liver due to CYP1A2, to later circulate to the lung, where they could be O-acetylated by NAT1 found in lung tissue.^58^ This metabolic activation pathway is supported by prior studies for 4-aminobiphenyl (ABP), which is a prototypical aromatic amine; ABP DNA adducts were detected in human lung tissues obtained by surgery or autopsy,^59^ as well as in human urinary bladder tissue biopsy samples and exfoliated urothelial cells.^60^^,^^61^ With regard to experimental animals, several studies indicate that exposure to BZ/BNA can increase the incidence of lung cancer; indeed, oral administration of BZ produced a high incidence of lung tumors in mice,^62^ intraperitoneal injection of BNA increased the incidence of lung adenomas in mice,^63^ and gavage administration of BNA caused lung tumor multiplicity in mice.^64^ The capacity of the human lung to metabolically activate BZ and BNA,^57^ the presence of DNA adducts of an aromatic amine in human lung tissues,^59^ and the experimental evidence of lung cancer for BZ and BNA in mice^62^^–^^64^ provide biological plausibility for the findings of this meta-analysis.

A major concern in interpreting our analysis was its inability to account for smoking, which is an important non-occupational risk factor for lung cancer.^65^ Although four studies obtained information on cigarette smoking, two of them calculated smoking-adjusted risk only for bladder cancer,^36^^,^^38^ and one of them had incomplete tobacco smoking data (lacking in about one third of cohort members).^10^ Only one study^11^ presented an effect estimate for lung cancer risk from a multivariate model; the relative risk for lung cancer incidence after adjustment for age at first exposure, smoking status, and other occupational co-exposure was 3.02 (95% CI, 0.84–10.93) for long duration of employment compared with the short duration group. Lack of adjustment for smoking might lead to overestimation of the association between lung cancer and exposure to BZ/BNA. Therefore, further studies with sufficient data on smoking are required for a better understanding of the risk for lung cancer in relation to occupational exposure to BZ/BNA.

A further limitation of our findings is the issue of heterogeneity between studies. Statistically significant heterogeneity was noted not only in the meta-analysis of all studies combined but also in subgroup analysis and sensitivity analysis. Heterogeneity is due to methodological diversity between the studies and shows up in the observed effects, being even far more different from each other than one would expect randomly.^66^ In observational epidemiology, study designs, populations, exposure assessing and outcome methods, and statistical analyses are rarely, if ever, the same.^50^ Therefore, meta-analysis of observational studies has the potential to introduce bias and confounding.^15^ Although we conducted subgroup analysis to explore sources of heterogeneity, study characteristics and study quality had little influence on our heterogeneity. A prior review based on 60 meta-analyses in occupational epidemiology pointed out that the most obvious finding was the various exposure definitions these studies employed and the lack of exposure data, which make comparisons onerous.^67^ The studies included in this meta-analysis also used a variety of BZ/BNA exposure surrogates, such as occupational groups, years of employment, and duration of employment. The differences in definitions of BZ/BNA exposure across studies might explain some of the heterogeneity between studies.

We are aware that occupational cohorts included in this study not only had simple exposures to BZ/BNA but had exposures to other chemicals as well. Although we conducted subgroup analyses and confirmed no difference in the lung cancer risk between workers with and without occupational exposure to known lung carcinogens, concomitant occupational exposures to other chemicals was not taken into consideration. Therefore, the results of this meta-analysis might be biased due to exposure to other chemicals.

Even though we systematically searched for all published cohort and case-control studies indexed in MEDLINE, EMBASE, and CINAHL, none of the identified studies were case-control studies. In general, case-control studies are superior to retrospective cohort studies regarding adequate control of confounding factors, including smoking.^68^ However, a main drawback of case-control studies is that exposure information is obtained by self-reporting after disease has occurred.^68^ Differential misclassification of exposure due to recall bias is likely, tending toward overestimation of the association between exposure and outcome. Therefore, including no case-control studies might prevent overestimation of the risk for lung cancer among workers exposed to BZ/BNA.

Despite these limitations, this study has the following strengths: 1) this is the first meta-analyses of lung cancer risk in workers exposed to BZ/BNA conducted according to the method of prospective meta-analysis, which means a meta-analysis of studies that were identified, evaluated, and determined to be eligible for the meta-analysis before the results of any of those studies became known^18^; 2) the total number of lung cancer deaths or incident cases was 1745 from all the cohort studies, providing summarized epidemiological evidence with adequate statistical power to examine the association between BZ/BNA exposure and lung cancer; and 3) our meta-analysis, restricted to workers with probable higher exposure, is reasonably consistent with an underlying dose-response effect.

In conclusion, this systematic review and meta-analysis suggests that occupational BZ/BNA exposure is associated with an increased risk of lung cancer. One important finding of this study is that pooled risk estimates were raised in the subgroup of studies in which workers were probably exposed to a high level of BZ/BNA. Although confounding by smoking cannot be completely ruled out, and the heterogeneity among studies requires cautious interpretation, our findings suggest that a lung cancer diagnosis following occupational BZ/BNA exposure may need to be considered a potential occupational disease.

## ONLINE ONLY MATERIALS

eTable 1. MEDLINE Search.

eTable 2. Cohort description and occupational exposure.

eTable 3. Study/cohort characteristics.

eTable 4. Related papers.

eTable 5. Results of quality assessment of the papers included in a meta-analysis of lung cancer and exposure to benzidine and/or beta-naphthylamine.

eTable 6. Risk estimates of studies included in analysis of highly exposed groups.

eFigure 1. Forest plot showing the influence of excluding each individual study on the pooled risk estimate (PRE) obtained using all studies for lung cancer among workers exposed to benzidine and/or beta-naphthylamine.

eFigure 2. Funnel plot of precision by log risk estimate.

Abstract in Japanese.
