# Supplementary material for: Risk of Lung Cancer in Workers Exposed to Benzidine and/or Beta-Naphthylamine: A Systematic Review and Meta-Analysis
Source: J Epidemiol. 2016 Sep 5;26(9):447–58. doi: 10.2188/jea.JE20150233 (PMC5008964; doi:10.2188/jea.JE20150233)
Supplement: eTable 1. [file je-26-447-s001.pdf]

eTable 1. MEDLINE search

#1	(benzidines OR benzidine) OR (biphenyldiamines OR biphenyldiamine) OR (bianilines OR bianiline) OR 2-naphthylamine OR beta-naphthylamine OR 2-aminonaphthalene OR dyestuff*[tiab]
#2	mortality OR incidence OR morbidity OR "cause of death" OR risk OR "adverse effects" OR epidemiology
#3	neoplasms OR carcinogen* OR cancer OR tumor OR tumour OR carcinoma
#4	work[tw] OR works*[tw] OR worka*[tw] OR worke*[tw] OR workg*[tw] OR worki*[tw] OR workl*[tw] OR workp*[tw]
#5	industr*[tiab] OR manufactur*[tiab] OR occupation*[tiab] OR employ*[tiab] OR product*[tiab] OR factory*[tiab] OR factories*[tiab]
#6	"occupational exposure"[MeSH] OR "occupational diseases"[MeSH]
#7	#4 OR #5 OR #6
#8	#1 AND #2 AND #3 AND #7
#9	"animals"[MeSH Terms:noexp]
#10	#8 NOT #9
