# Supplementary material for: Risk of Lung Cancer in Workers Exposed to Benzidine and/or Beta-Naphthylamine: A Systematic Review and Meta-Analysis
Source: J Epidemiol. 2016 Sep 5;26(9):447–58. doi: 10.2188/jea.JE20150233 (PMC5008964; doi:10.2188/jea.JE20150233)
Supplement: eTable 2. [file je-26-447-s002.pdf]

eTable 2. Cohort description and occupational exposure

Cohort group (ID)	First author	Cohort description	Occupational exposure to other chemicals
1	Fox	Total cohort of 41,981 men employed in the rubber and cablemaking industries on 1 February 1967, with one year or more employment duration. In this review, results are given for men employed in factories which had used BNA and who entered these factories before 1949 when BNA was used.	No description
2	Delzell 1982	Original cohort of 13,570 white male union members at a large rubber manufacturing facility for at least 2 years from 1940-71. In this study, the cohort comprised 2,666 men employed in the processing division where BNA was a possible contaminant in antioxidants.	No description
3	Morinaga 1982	Original cohort of 3,322 workers manufacturing BZ and BNA from 1950-78. A total of 244 had suffered from and died of cancer of genitourinary organs. This study investigated the organ sites of second primary cancers.	No description
4	Gustavsson	Total cohort of 12,212 workers employed for at least one year in two Swedish rubber manufacturing companies from 1930-75 (including white-collar workers as unexposed group, and excluding individuals who had terminated their employment before 1952). In this review, results are given for exposed male Swedish rubber workers first employed before 1951 when BNA was present in rubber antioxidants as an impurity (no description of the number, presumably about 3000).	No description
5	Costantini	Cohort of 2,926 men worked in the tanneries of the leather area for at least 6 months from 1950-81. BZ-based dyes were used in the tanning industry.	Chrome, o-dianisidine, o-tolidine, formaldehyde, chlorophenols
6	Delzell 1989	Total cohort of 2,642 men employed at a dye and resin manufacturing plant for at least 6 months from 1952-85. In this review, results are given for hourly employees (n=379) in the azo dye area where BZ was handled. Updated study (Sathiakumar 2000) did not described lung cancer risk amomg azo dye workers.	Dichlorobenzidine, o-dianisidine, o-tolidine
7	Sorahan	Total cohort of 36,691 men employed for at least one year in the rubber industries from 1946-60. In this review, results are given for workers first employed 1946-1950 when BNA was present in rubber antioxidants as an impurity.	No description
8	Chen	Cohort of 901 male workers exposed to the BZ-derived dyes for at least one year in the Shanghai leather tanning industry from 1961-65.	No description

Cohort group (ID)	First author	Cohort description	Occupational exposure to other chemicals
9	Morinaga 1990	Total cohort of 794 male workers who had been engaged in manufacturing and/or handling BZ and/or BNA from 1945-71 at two factories and who were alive in 1970. Only 604 men with identifiable vital status were considered for final analysis.	No description
10	You	Total cohort of 1,292 workers from seven factories producing BZ derived dyes from 1946-76. In this review, results are given for 550 men exposed to BZ for more than six months.	No description
11	Bulbulyan	Total cohort of 4,581 current employees of a dye production facility as of 1 January 1975. In this review, results are given for 514 men with jobs involving exposure to BZ or BNA for at least one month.	P-aminopenol
12	Naito	Total cohort of 442 individuals who worked at a dyestuff factory from 1935-88. Only 437 men were considered for final analysis.	Dianisidine, alpha-naphthylamine
13	Sitarek	Total cohort of 10,529 workers employed for at least 3 months in a plant producing different kinds of dyes using BZ from 1945-74. In this review, results are given for male workers exposed only to benzidine (subcohort I) and those exposed to benzidine and other occupational hazards (subcohort II) (no description of the number, presumably about 1500).	No description
14	Szeszenia-Dąbrowska	Cohort of 6,978 men employed for at least three months in a rubber goods factory from 1945-73. BNA was used from 1945 to 1953. The subcohort 1945-1953 was not separately analyzed. Information on comparability and assessment of outcome was confirmed by contacting the author.	Carbon black, silica, nitrosamine, asbestos, benzene, formaldehyde
15	Montanaro	Cohort of 1,244 workers (870 men and 374 women) employed at a chrome tannery from 1955-88 when workers were potentially exposed to BZ. This study did not show gender-specific SMR.	Chromium
16	Axtell	Cohort of 1,384 hourly employees who had worked at a synthetic dye plant that manufactured BNA from 1940-72. Only 1,314 men were considered for final analysis. Among the 1,314 subjects, 27 men were excluded from the analysis due to invalid information concerning date of birth, work history, or both, and 63 men were lost to follow-up. Therefore, the rate of loss to follow-up was 6.8% (90/1,314).	No description

Cohort group (ID)	First author	Cohort description	Occupational exposure to other chemicals
17	Cassidy	Total cohort of 400 (408 in the first publications of the cohort) employed at a chemical plant that produced or used BNA from 1940-81. In this review, results are given for 374 men.	Benzene, 2,3,6-trichlorophenylacetic acid, dichlorobenzene, arsenic, pentachlorophenol
18	Stern	Total cohort of 9,352 (9,365 in the original cohort) employed in two chrome tanneries from 1940-80. This study did not show gender-specific SMR. In this review, results are given for tanners who worked in the retan, color, and fat-liquoring department where benzidine-based samples were found (no description of the number, presumably about 2000).	Chrome, formaldehyde, dimethyl amine, ammonia, hydrogen sulfide, formic acid, sulfuric acid, toluene, acetone, xylene, butyl cellusolve, methyl isobutyl ketone
19	Rosenman	Total cohort of 538 workers employed at a single facility that produced BZ and dichlorobenzidine from 1960-77. In this review, results are given for 285 white men who worked from 1960-1972 when the facility produced BZ (this cohort could not exclude workers with non-exposure, such as office and nonproduction workers, because the cohort of workers was identified from Social Security Administration records).	Dichlorobenzidine, organophosphates, 4 di-n-propylamine-3,5-dintro-1-trifluorimethylbenzene
20	Mikoczy	Cohort of 2,027 tannery workers employed for at least one year from 1900-89 at three Swedish leather tanneries. Although published data did not show gender-specific SIR, we obtained male SIR for lung cancer by contacting the author.	Chrome, arsenic sulphides, methyl mercury, chlorophenols, formaldehyde
21	Pira	Original cohort of 906 men who had been employed since 1946, and had worked for at least 1year in a dyestuff factory from 1922-72 (1922-70 in the earlier studies). In this study, the cohort comprised 590 exposed workers (number of people who were exposed to BZ and/or BNA were 543). In subgroup analysis, this cohort was included in a national reference group.	Alpha-naphthylamine, fuchsin, o-toluidine
22	Brown	Total cohort of 997 individuals employed at a chemical manufacturing facility for at least 2 weeks from 1945-65 when BZ was produced. Only 847 men were considered for final analysis.	Dichlorobenzidine, o-dianisidine, o-tolidine
23	Tomioka	Total cohort of 243 individuals who were engaged in the production and/or use of BZ and/or BNA at a dyestuff factory from 1953-72. Only 224 men were considered for final analysis.	Bis(chloromethyl) ether, o-dianisidine
