# Supplementary material for: Risk of Lung Cancer in Workers Exposed to Benzidine and/or Beta-Naphthylamine: A Systematic Review and Meta-Analysis
Source: J Epidemiol. 2016 Sep 5;26(9):447–58. doi: 10.2188/jea.JE20150233 (PMC5008964; doi:10.2188/jea.JE20150233)
Supplement: eTable 3. [file je-26-447-s003.pdf]

eTable 3. Study/cohort characteristics

Cohort group (ID)	First author	Reference group	Cancer identification	Definition of lung cancer	Loss to follow-up	Period of employment	Follow-up period	Person-years	Total cancer Number of cases Risk estimate 95% CI	Bladder cancer Number of cases Risk estimate 95% CI
1	Fox	National	DC	ICD-8 162·1	2.7%	1967	1968 - 1974	NA	479<br>1.07<br>0.98-1.17	27<br>1.66<br>1.09-2.42
2	Delzell 1982	National	DC	ICD-7 162-163	NA	1940 - 1971	1940 - 1978	71,505	248<br>1.04<br>0.92-1.18	10<br>1.17<br>0.56-2.16
3	Morinaga 1982	Regional	CR	ICD-8 162	0%	1950 - 1978	1950 - 1978	2,001	12<br>1.57<br>0.81-2.75	NA
4	Gustavsson	National	DC and CR	ICD-8 162	3.0%	1930 - 1975	1959 - 1980	90,000	177<br>1.04<br>0.90-1.21	12<br>1.03<br>0.53-1.80
5	Costantini	National	Official records of the municipalites	ICD-8 162	0.1%	1950 - 1981	1950 - 1983	44,033	85<br>0.97<br>0.77-1.19	5<br>1.50<br>0.48-3.49
6	Delzell 1989	National	DC	ICD-8<br>no code	3.6%	1952 - 1985	1952 - 1985	NA	16<br>1.93<br>1.10-3.12	2<br>12.50<br>1.51-45.13
7	Sorahan	National	DC	ICD-8 162-163	3.3%	1946 - 1960	1946 - 1985	NA	3,344[d]<br>1.13<br>1.09-1.17	106[d]<br>1.00<br>0.81-1.19
8	Chen	Regional	NA	NA	4.1%	1961 - 1965	1966 - 1985	NA	86<br>0.96<br>0.77-1.19	6<br>2.73<br>1.00-5.95
9	Morinaga 1990	Regional	DC	ICD-8 162	24.1%	1945 - 1970	1970 - 1986	9,709	35<br>1.01<br>0.70-1.40	10<br>12.20<br>5.86-22.45

Cohort group (ID)	First author	Reference group	Cancer identification	Definition of lung cancer	Loss to follow-up	Period of employment	Follow-up period	Person-years	Total cancer Number of cases Risk estimate 95% CI	Bladder cancer Number of cases Risk estimate 95% CI
10	You	Regional	MD	NA	NA	1946 - 1976	1946 - 1982	10,744	44<br>1.34<br>0.97-1.80	14<br>19.18<br>10.47-32.22
11	Bulbulyan	Regional	CR	ICD-8 161-162	0%	1930 - 1975	1975 - 1989	5,470	86<br>2.05<br>1.65-2.55	19<br>10.82<br>6.51-16.88
12	Naito	National	ND	ICD-9 162	0%	1935 - 1972	1935 - 1992	14,938	49<br>1.32<br>0.98-1.75	18<br>38.25<br>22.68-60.44
13	Sitarek	National	NA	ICD-9 162	NA	1945 - 1974	1945 - 1991	NA	80<br>1.54<br>1.23-1.92	24<br>15.68<br>10.05-23.21
14	Szeszenia-Dąbrowska	National	DC	ICD-9 162	11%	1945 - 1973	1945 - 1990	NA	421<br>1.08<br>0.98-1.18	16<br>1.27<br>0.73-2.06
15	Montanaro	National	DC	ICD-9 162	4.3%	1955 - 1988	1955 - 1994	36,414	123<br>1.12<br>0.94-1.34	10<br>2.42<br>1.16-4.46
16	Axtell	National[a]	DC	NA[c]	6.8%	1940 - 1972	1940 - 1992	32,481	92<br>1.30<br>1.05-1.60	3<br>2.39<br>0.49-6.99
17	Cassidy	Regional	DC	NA[c]	2.7%	1940 - 1981	1960 - 1998	8,665	28<br>3.08<br>2.05-4.46	4<br>16.83<br>4.59-43.10
18	Stern	National[a]	DC	ICD-9 162	4.3%	1940 - 1980	1940 - 1993	NA	126<br>0.93<br>0.78-1.11	2<br>0.58<br>0.07-2.09

Cohort group (ID)	First author	Reference group	Cancer identification	Definition of lung cancer	Loss to follow-up	Period of employment	Follow-up period	Person-years	Total cancer Number of cases Risk estimate 95% CI	Bladder cancer Number of cases Risk estimate 95% CI
19	Rosenman	National	CR and DC	NA	0.0%	1960 - 1977	1960 - 2001	NA	25<br>1.52<br>0.98-2.24	3<br>9.62<br>1.98-28.12
20	Mikoczy	National	CR	ICD-7 162·1	0.6%	1900 - 1989	1958 - 1999	56,022	351<br>1.16<br>1.04-1.29	24<br>0.96<br>0.61-1.42
21	Pira	National[b] 1951-1980, Regional 1981-2003	DC	NA	13.9%	1922 - 1970	1946 - 2003	17,754	163<br>2.11<br>1.80-2.46	56<br>16.45<br>12.42-21.36
22	Brown	Regional	CR, DC, and MR	NA	NA	1945 - 1965	1945 - 2005	31,678	230<br>0.84<br>0.74-0.96	25<br>1.12<br>0.73-1.66
23	Tomioka	National[a]	CR, DC, and MR	ICD-10 C33-34	3.6%	1953 - 1972	1973 - 2011	7,668	81<br>1.58<br>1.26-1.98	7<br>4.70<br>1.89-9.67

CI, confidence interval; CR, cancer registry; DC, death certificate; ICD, International Classification of Diseases; MR, medical record; NA, not available.

[a]Although the study used both national and regional populations to compute the expected deaths/cases, results based on the national statistics were used, because regional rates were not available for the entire follow-up duration

[b]Subgroup analyses used a national reference due to a longer period of adaptation

[c]All deaths were coded to underlying cause according to the ICD rules in effect at the time of the death

[d]Data of total cohort were used due to a lack of subcohort data
