# Supplementary material for: Risk of Lung Cancer in Workers Exposed to Benzidine and/or Beta-Naphthylamine: A Systematic Review and Meta-Analysis
Source: J Epidemiol. 2016 Sep 5;26(9):447–58. doi: 10.2188/jea.JE20150233 (PMC5008964; doi:10.2188/jea.JE20150233)
Supplement: eTable 4. [file je-26-447-s004.pdf]

eTable 4. Related papers

Cohort group (ID)	First author	Related papers
1	Fox	Fox AJ, Lindars DC, Owen R. A survey of occupational cancer in the rubber and cablemaking industries: results of five-year analysis, 1967-71. Br J Ind Med. 1974;31(2):140-51.
2	Delzell 1982	Monson R, Nakano K. Mortality among rubber workers. I. White male union employees in Akron, Ohio. Am J Epidemiol 1976; 103: 284–296.
		Monson RR and Fine LJ. Cancer mortality and morbidity among rubber workers. J Natl Cancer Inst 1978; 61: 1047-1053.
		Delzell E, Monson R. Mortality among rubber workers. III. Cause-specific mortality, 1940-1978. J Occup Med 1981; 23: 677-684.
3	Morinaga 1982	No
4	Gustavsson	Holmberg B, Westerholm P, Maasing R, Kestrup L, Gumaelius K, Holmlund L, Englund A. Retrospective cohort study of two plants in the Swedish rubber industry. Scand J Work Environ Health. 1983;9 Suppl 2:59-68.
5	Costantini	No
6	Delzell 1989	Sathiakumar N, Delzell E. An updated mortality study of workers at a dye and resin manufacturing plant. J Occup Environ Med. 2000;42(7):762-71.
7	Sorahan	Sorahan T, Parkes HG, Veys CA, Waterhouse JA Cancer mortality in the British rubber industry: 1946-80. Br J Ind Med. 1986;43(6):363-73.
		Parkes HG, Veys CA, Waterhouse JA, Peters A. Cancer mortality in the British rubber industry. Br J Ind Med. 1982;39(3):209-20.
8	Chen	No
9	Morinaga 1990	Morinaga K, Hara I, Yutani S, Sera Y. Uses of population-based cancer registration in occupational epidemiology: experience in Osaka. J UOEH. 1983;5 Suppl:215-23.
10	You	No
11	Bulbulyan	No
12	Naito	No
13	Sitarek	No

Cohort group (ID)	First author	Related papers
14	Szeszenia-Dąbrowska	Szeszenia-Dąbrowska N, Wilczyńska U, Szymczak W, Kaczmarek T. Mortality among rubber industry workers. Ⅰ. general mortality. Med Pr 1990;41:326-334.
		Szeszenia-Dąbrowska N, Wilczyńska U, Kaczmarek T, Szymczak W. Cancer mortality among male workers in the Polish rubber industry. Pol J Occup Med Environ Health. 1991;4(2):149-57.
15	Montanaro	Puntoni R, Valerio A, Cresta E, Filiberti R, Bonassi S. Vercelli M. Studio di mortality fra i lavoratori di una conceria. MedLav 1984;75:471-7.
16	Axtell	Schulte PA, Ringen K, Hemstreet GP, Altekruse EB, Gullen WH, Patton MG, Allsbrook WC, Crosby JH, West SS, Witherington R, Koss L, Bales CE, Tillet S, Rooks SCF, Stern F, Stringer W, Schmidt VA, Brubaker MM. Risk assessment of a cohort exposed to aromatic amines. J Occup Med 1985; 27:115-121.
		Schulte PA, Ringen K, Hemstreet GP, Altekruse EB, Gullen WH, Tillett S, Allsbrook WC, Crosby JH, Witherington R, Stringer W, Brubaker MM. Risk factors for bladder cancer in a cohort exposed to aromatic amines. Cancer 1986;58:2156-2162.
		Stern FB, Murthy LI, Beaumont JJ, Schulte PA, Halperin WE. Notification and risk assessment for bladder cancer of a cohort exposed to aromatic amines. III. Mortality among workers exposed to aromatic amines in the last beta-naphthylamine manufacturing facility in the United States. J Occup Med. 1985;27(7):495-500.
17	Cassidy	Marsh GM, Leviton LC, Talbott EO, Callahan C, Pavlock D, Hemstreet G, Logue JN, Fox J, Schulte P. Drake Chemical Workers' Health Registry Study: I. Notification and medical surveillance of a group of workers at high risk of developing bladder cancer. Am J Ind Med.
18	Stern	Stern FB, Beaumont JJ, Halperin WE, Murthy LI, Hills BW, Fajen JM. Mortality of chrome leather tannery workers and chemical exposures in tanneries. Scand J Work Environ Health. 1987;13(2):108-17.
19	Rosenman	No
20	Mikoczy	Mikoczy Z, Schütz A, Hagmar L. Cancer incidence and mortality among Swedish leather tanners. Occup Environ Med. 1994;51(8):530-5.
21	Pira	Rubino GF, Scansetti G, Piolatto G, Pira E. The carcinogenic effect of aromatic amines: an epidemiological study on the role of o-toluidine and 4,4'-methylene bis (2-methylaniline) in inducing bladder cancer in man. Environ Res. 1982;27(2):241-54.
		Decarli A, Peto J, Piolatto G, La Vecchia C. Bladder cancer mortality of workers exposed to aromatic amines: analysis of models of carcinogenesis. Br J Cancer. 1985;51(5):707-12.
		Piolatto G, Negri E, La Vecchia C, Pira E, Decarli A, Peto J. Bladder cancer mortality of workers exposed to aromatic amines: an updated analysis. Br J Cancer. 1991;63(3):457-9.
22	Brown	Meigs JW, Marrett LD, Ulrich FU, Flannery JT. Bladder tumor incidence among workers exposed to benzidine: a thirty-year follow-up. J Natl Cancer Inst. 1986;76(1):1-8.
23	Tomioka	No
