# Supplementary material for: Risk of Lung Cancer in Workers Exposed to Benzidine and/or Beta-Naphthylamine: A Systematic Review and Meta-Analysis
Source: J Epidemiol. 2016 Sep 5;26(9):447–58. doi: 10.2188/jea.JE20150233 (PMC5008964; doi:10.2188/jea.JE20150233)
Supplement: eTable 5. [file je-26-447-s005.pdf]

eTable 5. Results of quality assessment of the papers included in a meta-analysis of lung cancer and exposure to benzidine and/or beta-naphthylamine

Cohort group (ID)	First author	Quality assessment component				
		Representativeness of the exposed cohort	Exposure	Comparability	Assessment of outcome	Adequacy of follow-up
1	Fox	Representative	Formal	ND	Formal	Virtually complete
2	Delzell	Representative	Formal	ND	Formal	ND
3	Morinaga	ND	Formal	ND	Formal	ND
4	Gustavsson	Representative	Formal	Standard	Formal	Virtually complete
5	Costantini	Representative	ND	Standard	ND	Virtually complete
6	Delzell	Representative	Formal	ND	Formal	Virtually complete
7	Sorahan	Representative	Formal	Standard	Formal	Virtually complete
8	Chen	Representative	Formal	ND	Formal	Virtually complete
9	Morinaga	Representative	Formal	Standard	Formal	Adequate
10	You	Representative	Formal	ND	ND	ND
11	Bulbulyan	Representative	Formal	Standard	Formal	ND
12	Naito	Representative	Formal	ND	ND	Virtually complete
13	Sitarek	Representative	Formal	ND	ND	Inadequate
14	Szeszenia-Dąbrowska	Representative	Formal	Standard[a]	Formal[a]	Inadequate[a]
15	Montanaro	Representative	Informal	Standard	Formal	Virtually complete
16	Axtell	Representative	Formal	Standard	Formal	Inadequate
17	Cassidy	Unrepresentative	Informal	Standard	Formal	Virtually complete
18	Stern	Representative	Formal	Standard	Formal	Virtually complete
19	Rosenman	Representative	Informal	Standard	Formal	ND
20	Mikoczy	Unrepresentative	Formal	Nonstandard[a]	Formal	Virtually complete
21	Pira	Representative	Formal	Nonstandard	Formal	Adequate
22	Brown	Representative	Formal	Standard	Formal	ND
23	Tomioka	Representative	Formal	Nonstandard	Formal	Virtually complete

ND, no description.

[a]Confirmed by contacting author
