# Supplementary material for: Risk of Lung Cancer in Workers Exposed to Benzidine and/or Beta-Naphthylamine: A Systematic Review and Meta-Analysis
Source: J Epidemiol. 2016 Sep 5;26(9):447–58. doi: 10.2188/jea.JE20150233 (PMC5008964; doi:10.2188/jea.JE20150233)
Supplement: eTable 6. [file je-26-447-s006.pdf]

eTable 6. Risk estimates of studies included in analysis of highly exposed groups

Reference	Year of publication	Number of lung cancer cases	Risk estimate	95% CI	Variable (highest category)
Gustavsson	1986	4	2.04	0.66 - 6.28	Long duration of exposure (≥5 years), a latency of at least 20 years, and first employment before 1951
You	1990	5	1.87	0.70 - 5.00	Workers engaged in jobs of synthetic reaction
Montanaro	1997	13	0.89	0.49 - 1.61	Long duration of employment (≥15 years)
Axtell	1998	6	5.09	2.09 - 12.40	Long duration of employment (≥10 years)
Cassidy	2003	5	3.19	1.19 - 8.54	Year of hire (<1963) or duration of employment (≥5 years)
Tomioka	2015	15	3.31	1.93 - 5.68	Long duration of employment (≥8.3 years)
