# Supplementary material for: Risk of Lung Cancer in Workers Exposed to Benzidine and/or Beta-Naphthylamine: A Systematic Review and Meta-Analysis
Source: J Epidemiol. 2016 Sep 5;26(9):447–58. doi: 10.2188/jea.JE20150233 (PMC5008964; doi:10.2188/jea.JE20150233)
Supplement: eFigure 1. [file je-26-447-s007.pdf]

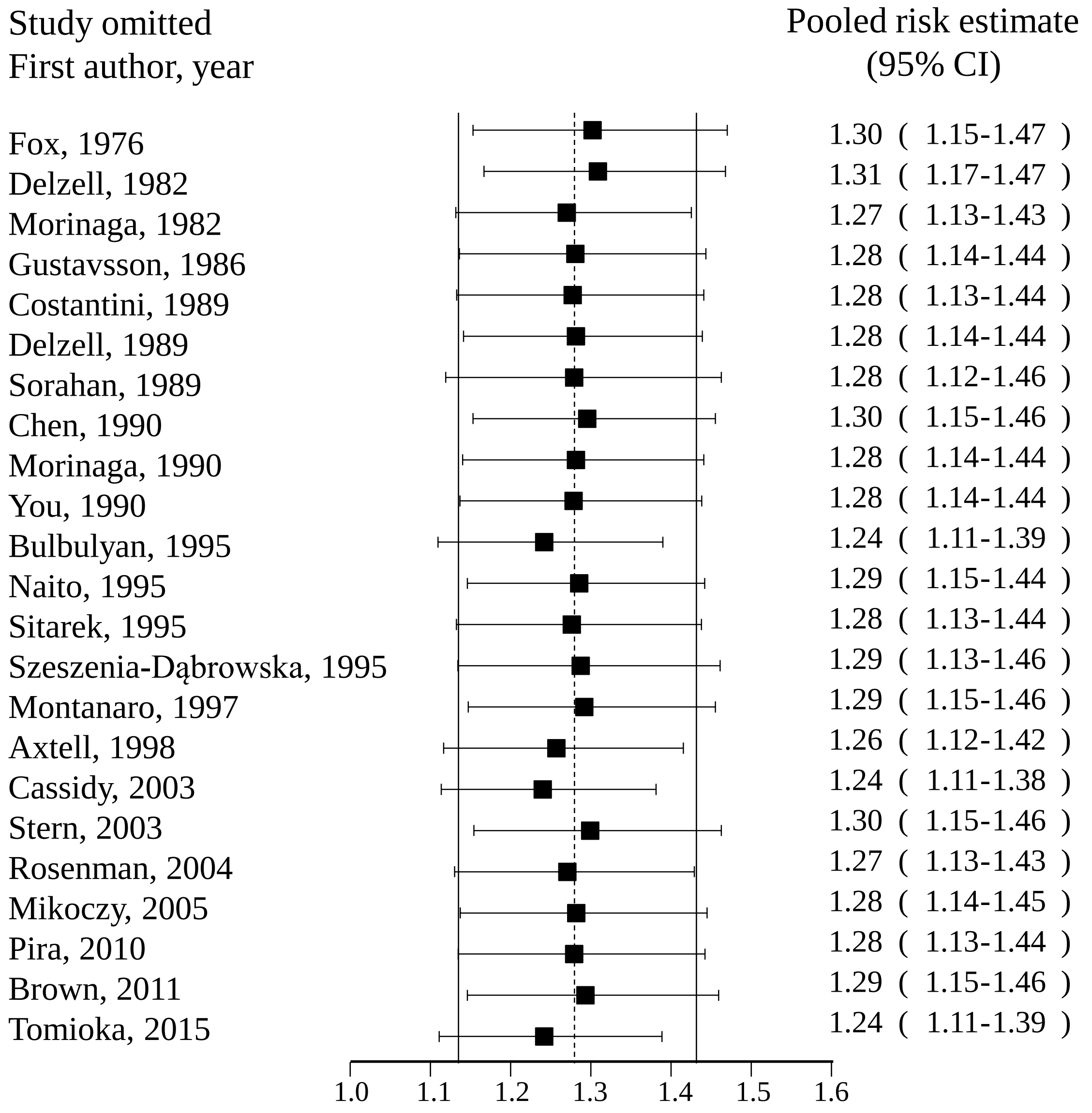

eFigure 1. Forest plot showing the influence of excluding each individual study on the pooled risk estimate (PRE) obtained using all studies for lung cancer among workers exposed to benzidine and/or beta-naphthylamine. Vertical dashed line = PRE obtained using all studies; vertical solid lines = 95% CI of the PRE using all studies; block with line = PRE with 95% CI obtained by omitting the mentioned study. CI, confidence interval.
