# Supplementary figures and images for: Risk of Lung Cancer in Workers Exposed to Benzidine and/or Beta-Naphthylamine: A Systematic Review and Meta-Analysis
Source: J Epidemiol. 2016 Sep 5;26(9):447–58. doi: 10.2188/jea.JE20150233 (PMC5008964; doi:10.2188/jea.JE20150233)

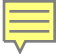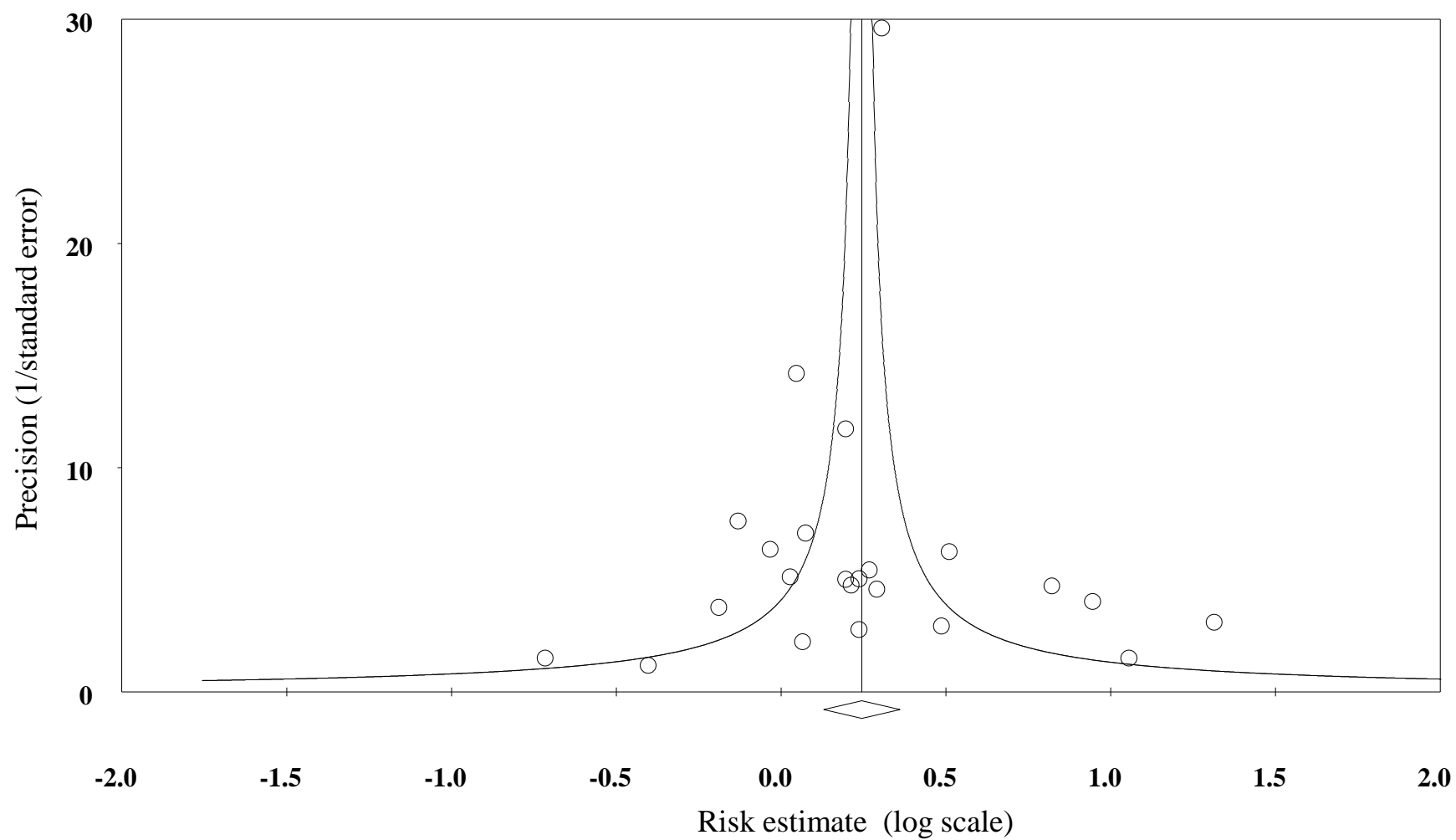

**eFigure 2.** Funnel plot of precision by log risk estimate

Supplement: eFigure 2. [file je-26-447-s008.pdf]
