# Supplementary material for: Risk of Lung Cancer in Workers Exposed to Benzidine and/or Beta-Naphthylamine: A Systematic Review and Meta-Analysis
Source: J Epidemiol. 2016 Sep 5;26(9):447–58. doi: 10.2188/jea.JE20150233 (PMC5008964; doi:10.2188/jea.JE20150233)
Supplement: Abstract in Japanese. [file je-26-447-s009.pdf]

ベンジジン、β‐ナフチルアミンに曝露された労働者の肺癌リスク：システマティック・レビューおよびメタ･アナリシス

冨岡公子[1]、佐伯圭吾[2]、大林賢史[2]、車谷典男[1,2]

[1]奈良県立医科大学県民健康増進支援センター
[2]奈良県立医科大学地域健康医学教室

ベンジジン、β‐ナフチルアミンは国際がん研究機関によって人に膀胱癌を引き起こす明らかな発がん物質であると分類されている。しかし、ベンジジン、β‐ナフチルアミンの曝露と肺癌との関連については十分検討されていない。我々はベンジジン、β‐ナフチルアミンに曝露された労働者における肺がんリスクを評価することを目的にシステマティック・レビューおよびメタ･アナリシスを行った。職業性ベンジジン、β‐ナフチルアミン曝露と関心のあるアウトカム（肺がんによる死亡や罹患）を報告した研究を同定するために系統的文献検索を実施した。メタ･アナリシスはランダム効果モデルを用いて標準化死亡比や標準化罹患比を結合させた。1,745 の肺がん症例を含む 23 の歴史的コホート研究を同定した；喫煙を調整した肺がんリスクを報告していた研究は 1 つだけであった。すべての研究を結合することによって、有意な異質性を伴った（$I^2 = 64.1\%$, $p <0.001$）有意な肺がんリスクの上昇が認められた（pooled SMR/SIR 1.28; 95% CI, 1.14‐1.43）。効果の推定値はベンジジン、β‐ナフチルアミンの直接曝露を伴った研究（すなわち、染料や製造産業）（pooled SMR/SIR 1.58; 95% CI, 1.31‐1.89）やベンジジン、β‐ナフチルアミンに関連した膀胱癌の SMR/SIR が 4.7 以上の研究（pooled SMR/SIR 1.68; 95% CI, 1.35‐2.09）で高くなっていた。効果の推定値はクロム、アスベスト、ヒ素、ビスクロロメチルエーテルの職業性曝露を伴っている研究と伴っていない研究では同じであった。累積メタ・アナリシスを行った結果、職業性ベンジジン、β‐ナフチルアミン曝露と肺癌との関連は 1995 年時点で確立していた。このメタ･アナリシスの結果は喫煙や異質性によって交絡されている可能性があるが、我々の発見は職業性ベンジジン、β‐ナフチルアミン曝露を伴う肺癌は職業癌の可能性を考慮すべきであることを示唆している。

キーワード：ベンジジン、β‐ナフチルアミン、肺癌、職業性曝露、システマティック・レビュー
